# Gender Differences in Prefrontal Cortex Response to Negative Emotional Stimuli in Drivers

**DOI:** 10.3390/brainsci14090884

**Published:** 2024-08-30

**Authors:** Ferran Balada, Anton Aluja, Óscar García, Neus Aymamí, Luis F. García

**Affiliations:** 1 Lleida Institute for Biomedical Research, Dr. Pifarré Foundation, 25198 Lleida, Spain; ferran.balada@uab.cat (F.B.); oscar.garcia@universidadeuropea.es (Ó.G.); naymami@gss.cat (N.A.); luis.garcia@uam.es (L.F.G.); 2 Departamento de Psicobiologia i Metodología CCSS, Facultad de Psicologia, Autonomous University of Barcelona, 08193 Barcelona, Spain; 3 Departamento de Psicología, Faculdat de Psicología, University of Lleida, 25001 Lleida, Spain; 4 Department of Psychology, European University of Madrid, 28670 Madrid, Spain; 5 Psychiatry, Mental Health and Addictions Service, Santa Maria Hospital of Lleida, 25198 Lleida, Spain; 6 Departamento de Psicología Biológica y de la Salud, Facultad de Psicología, Autonomous University of Madrid, 28049 Madrid, Spain

**Keywords:** fNIRs, driving adaptive style, prefrontal cortex, emotions

## Abstract

Background: Road safety improvement is a governmental priority due to driver-caused accidents. Driving style variation affects safety, with emotional regulation being pivotal. However, functional near-infrared spectroscopy (fNIRS) studies show inconsistent prefrontal cortex activity during emotion processing. This study examines prefrontal cortex response to negative emotional stimuli, particularly traffic accident images, across drivers diverse in age and gender. Method: The study involved 118 healthy males (44.38 ± 12.98 years) and 84 females (38.89 ± 10.60 years). The Multidimensional Driving Style Inventory (MDSI) was used to assess driving behavior alongside fNIRS recordings. Participants viewed traffic accident and neutral images while prefrontal oxygenation was monitored. Results: Women rated traffic accidents (*t*-test = 2.43; *p* < 0.016) and neutral images (*t*-test = 2.19; *p* < 0.030) lower in valence than men. Arousal differences were significant for traffic accident images (*t*-test = −3.06; *p* < 0.002). correlational analysis found an inverse relationship between Dissociative scale scores and oxygenation (all *p*-values ≤ 0.013). Greater prefrontal oxygenation occurred with neutral images compared to traffic accidents. Left hemisphere differences (*t*-test = 3.23; *p* < 0.001) exceeded right hemisphere differences (*t*-test = 2.46; *p* < 0.015). Subgroup analysis showed male participants to be driving these disparities. Among adaptive drivers, significant oxygenation differences between neutral and accident images were evident in both hemispheres (left: *t*-test = 2.72, *p* < 0.009; right: *t*-test = 2.22, *p* < 0.030). Conclusions: Male drivers with maladaptive driving styles, particularly dissociative ones, exhibit reduced prefrontal oxygenation when exposed to neutral and traffic accident images. This response was absent in female drivers, with no notable age-related differences.

## 1. Introduction

Improving road safety is a priority concern of all governments. United Nations Resolution 74/299, adopted in 2020, establishes a target of reducing road traffic fatalities and injuries by 50% within the decade spanning from 2021 to 2030 [1]. Road crashes can be attributed to various factors, including environmental conditions, vehicle specifications, and human behaviors. Research suggests that drivers cause up to 94% of accidents [2]. 

Elander et al. [3] proposed a distinction between driving skills and driving styles, with the former encompassing experiential and attentional factors, while the latter involves choices such as driving speed, adherence to traffic regulations, and gap acceptance. Several questionnaires have been developed to assess driving styles [4,5,6,7]. The Multidimensional Driving Style Inventory (MDSI) has emerged as an effective tool for examining both adaptive and maladaptive driving behaviors [8].

Drivers with maladaptive driving styles are characterized by high speeds, aggressive behavior, and poor vehicle control. Anger, high driving motivation, and being male are predictors of this driving style [9]. In fact, research shows that drivers with maladaptive driving styles tend to have difficulty regulating their emotions compared to drivers with adaptive driving styles [10,11]. Negative emotional states have been shown to reduce the perception of danger [12] and increase driving errors [13,14], thereby promoting risky driving behaviors [15]. Conversely, positive emotional states have also been linked to risky driving tendencie [16]. Viewing pleasant images significantly decreased speed in a simulator relative to viewing neutral or unpleasant images in a sample of 72 men and women [17]. Also, viewing a clip showing a hazard after viewing a neutral image elicited a higher risk assessment, a greater physiological response, and a longer duration of gaze fixation than positive or negative valence images [18]. The difficulty in regulating emotions appears to be a contributing factor in the manifestation of maladaptive driving styles, particularly among young individuals [11]. The adoption of emotion regulation techniques, particularly reappraisal, has been shown to promote more adaptive driving styles [19], although some studies have shown more adaptive driving in the reappraisal-down condition but not in the reappraisal-up condition [20]. In a recent systematic review, Pizzo et al. [21] found that the use of these cognitive techniques could reduce risky driving in young people. Moreover, task-focused coping strategies, such as emotion-focused coping strategies, have been shown to be effective in complying with speed limits, although gender differences have been found [17].

The prefrontal cortex plays a crucial role in the reappraisal process. The ventral areas of the prefrontal cortex evaluate the emotional characteristics of stimuli, while the dorsal areas regulate these emotional responses [22,23,24,25]. Electrophysiological studies support the involvement of the prefrontal cortex in emotional regulation. When exposed to unpleasant images accompanied by a tone, activation in the orbicularis oculi muscle, which produces the startle response, is increased [26,27,28]. Emotional regulation has been found to attenuate this response [29], particularly in the left prefrontal areas [30]. Several studies have shown gender differences in the brain’s response to emotions. It is noticeable that greater lateralization is observed in males [31].

In recent years, there has been growing interest in functional near-infrared spectroscopy (fNIRS) studies. This technique allows imaging in a more user-friendly environment, with good temporal resolution and low cost. However, it has limitations, such as the detection of surface structures and low spatial resolution [32]. Recent systematic reviews of fNIRS studies measuring neural activation during emotion processing in healthy individuals have shown contradictory findings. Westgarth et al. [33], in a recent systematic review, found increased prefrontal activity during emotional regulation in 9 out of 11 studies. In two studies, it was only observed during the regulation of negative stimuli, while studies related to perception or emotional experience showed more inconsistent results. Similar results can be found in other reviews [34,35]. 

Several factors may contribute to these inconsistent results, including sample characteristics, stimulus type, and task demands [33]. In the extensive review by Westgarth et al. [33], most of the evaluated studies focused on young adults (53/72; Mean age = 23.4 years). Among the 16 studies involving middle-aged adults (mean age = 35.9 years), the maximum sample size was 28 participants, and only in three studies was there a balanced participation by gender. Moreover, the majority (6/16) of these 16 studies with middle-aged populations used face stimuli, and only a few utilized emotionally regulated imageries. Several studies have indicated diminished resting prefrontal brain activity in younger adults [36]. However, differences between adults and younger individuals diminish in scenarios such as driving [37]. Conversely, older adults exhibit lower prefrontal activity during emotional reappraisal tasks [38]. The aim of this study is to investigate how different driving styles, particularly maladaptive ones characterized by high speeds and aggressive behavior, may influence the regulation of emotions in the prefrontal cortex. For this purpose, we will evaluate the response to neutral and negative valence images related to traffic accidents in a large sample of male and female drivers in our environment. The present study is eminently exploratory in nature. Nonetheless, based on the data presented above, we can hypothesize some results. Specifically, difficulties in emotion regulation in drivers with maladaptive driving styles should involve reduced activity in the prefrontal cortex involved in emotional regulation. This is why we expect to find that those drivers with more maladaptive styles (characterized by high scores on the Recklessness, Anger, Anxiety, and Dissociative scales and low scores on the Carefulness and Distress Reduction scales) will present lower prefrontal activation after exposure to imagery, especially in the left hemisphere of the prefrontal cortex. This lateralization might be more evident in the case of male and older participants.

## 2. Method

### 2.1. Participants and Procedure

One hundred and eighteen healthy males (44.38 ± 12.98 years) and eighty-four healthy females (38.89 ± 10.60 years) participated in this study. Participants were recruited from a collective email addressed to the teaching and administration staff of the university. They received an incentive of 25 euros. The participants had a driving license and stated that they drive regularly. The study was conducted in a midsize city in a rural area where daily displacements are frequent. Participants declared no history of substance abuse. The phase of the menstrual cycle and oral contraceptive intake in women were monitored, as well as the hours of sleep (mean sleep < 7.1 ± 1.0) of each participant. They were also instructed not to consume stimulants or tobacco in the previous 12 h. In total, four blocks of 6 images each were presented in a pseudo-random manner. Two of these blocks corresponded to neutral pictures, and the other two to pictures related to traffic accidents. Before the presentation of each block, the word “Look” appeared for 2 s, and then the different images were presented for 6 s each, with an ITI varying between 0.5 and 1 s (mean 0.75). The presentation of the pictures in the same block was randomized. The total duration of each block was 40.5 s. After the end of the image presentation, a screen appeared that allowed the valence and arousal characteristics of the block to be rated on a scale of 0 to 100. Afterwards, a period of 10 s was established to return to basal brain activation levels before the presentation of the next block. The study was authorized by the ethics committee. All participants signed an informed consent.

### 2.2. Material

#### 2.2.1. Multidimensional Driving Style Inventory (MDSI)

The MDSI was developed to evaluate different driving styles [7] and validated in Spain [39]. This questionnaire has 34 items and considers adaptive and maladaptive driving styles. The response format is a 6-point Likert type ranging from “*not at all*” (1) to “*very much*” (6). Careful and Distress reduction are the adaptive styles, whereas Reckless, Angry, Anxious, and Dissociative are the maladaptive styles. In the original article, internal consistency ranged from 0.72 to 0.86, and in the Spanish version, between 0.65 and 0.80.

#### 2.2.2. Pictures

**Figure 1:**
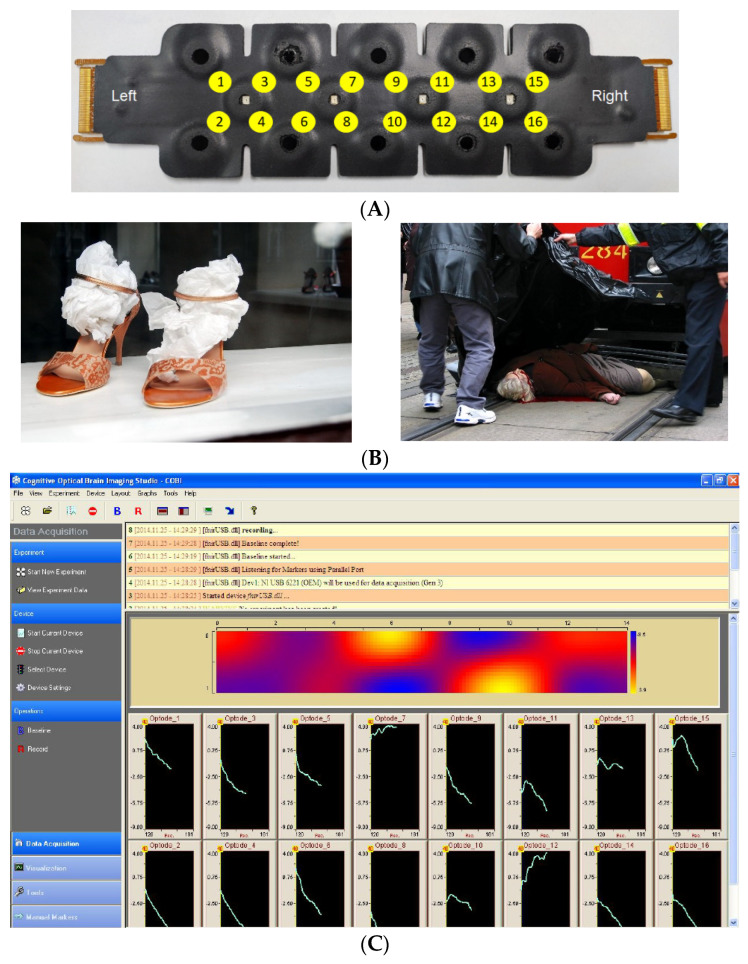
(**A**) Sensor used for fNIR recording with indication of the channels. (**B**) Example of images of each of the four blocks presented. On the left are the neutral valence images. On the right are images related to traffic accidents. (**C**) Screenshot of the fNIR signal-recording software used to obtain the data. The lower part of the image shows the register corresponding to each of the 16 channels analysed.

We selected 24 authorized pictures from the International Affective Picture System (IAPS) [40]. The IAPS labels and codes for the stimuli used in the first neutral block were: Spoon (7004), Stool (7025), Clock (7211), File cabinets (7224), Chair (7235), and Cabinet (7705) and the Nencki Affective Picture System (NAPS) [41]. The block of neutral images was composed of Landscapes_045_h, Landscapes_091_h, Objects_166_h, Objects_204_h, Objects_237_h, and Objects_307_v. The first block of accidents was composed of the following images: People_004_h, People_007_h, People_009_h, People_010_h, People_013_v, and People_016_h. Finally, the other block of accident images included People_020_h, People_021_h, People_022_h, People_226_h, Objects_003_h, and Objects_149_h. (NAPS) databases (Figure 1B). Pictures were grouped into four blocks of six images each. Two of these blocks corresponded to neutral images, while the other two blocks were related to traffic accidents. With the aim of evaluating the valence and arousal of the images used, at the end of each block, the participants were asked to indicate using semantic differential scales to indicate whether the images presented in that block had caused them feelings of being happy-sad, angry-glad, exalted-calm or indifferent-impressed.

#### 2.2.3. fNIR Recording and Data Analysis

The recordings took place within a Faraday cage setup consisting of two compartments with electromagnetic and acoustic isolation. One compartment was designated for the experimenter, while the other was for the participant. The participant’s area featured a 32-inch SONY screen for image presentation, with the participant seated in a chair with armrests positioned 100 cm away from the screen. Indirect lighting was present in the participant’s space, situated behind the monitor and utilizing a strip of LEDs for illumination. The fNIR module used allows us to monitor changes in prefrontal blood oxygenation during picture presentation. These changes are detected by means of a sensor featuring 4 LED light sources and 10 photodetectors that allow us to obtain the changes in oxygenated (O2Hb) and deoxygenated (HHb) hemoglobin (measured in μmol/L) in 16 channels by combining the signals captured by the photodetectors (Figure 1A). The raw data obtained were visually evaluated to remove channels with excessive saturation (higher than 4000 mV) or lack of saturation (lower than 400 mV). For the valid records, a Finite Impulse Response (FIR) filter with an order of 20 and a cutoff frequency of 0.1 was implemented to reduce high-frequency noise and breathing and respiratory effects. Following this, a Sliding-window Motion Artefact Rejection (SMAR) algorithm was utilized to eliminate artifacts and saturated channels. Finally, the Modified Beer–Lambert law was applied along with a linear trend reduction to constrain signal deviations. The difference between oxygenated and deoxygenated hemoglobin indicates the oxygenation (Oxy) of that area. Sensor placement is according to electrode positions F7, FP1, FP2, and F8, International EEG System 10–20, corresponding to Brodmann areas 9, 10, 45, 46, and 47. Before the start of the task, the light intensity and amplification of each channel were calibrated to avoid weak or saturated signals. Recording was performed using the COBI data collection suite (Biopac System Inc. 42 Aero Camino, Goleta, California, United States; Figure 1C), as described [42]. The fNIRS software (Version 4.11.7139.36128) was used to analyze the data obtained [43] (Figure 1C).

#### 2.2.4. Statistical Analysis

The values of skewness and kurtosis of the variables were analyzed. Skewness values ranged from −1.36 to 1.03. Kurtosis values ranged from −0.91 to 1.52, except for the Careful scale, where a value of 4.25 was obtained. A principal component analysis of an unrotated factor was carried out using all MDSI scales to have a single value for driving styles. This total score can be computed as the overall driving style. Note that Angry, Reckless, Dissociative, and Anxious scales load positive (all > 0.50), and Careful loads negative (−0.48), while Distress Reduction scores virtually zero on this factor. The factor score showed a strong positive correlation (0.35; *p* < 0.001) with the number of traffic fines. Outlier detection was performed using Tukey’s nonparametric fence method [44], which is based on the interquartile range. The formula Q3 + k (Q3 − Q1) was used to calculate the outer fence, where k corresponds to a constant value of 3.

The participants were divided into three groups (low, medium, and high maladaptive driving style), using the 33rd (−0.545) and 66th (0.300) percentiles of the factor obtained as cut-off points. In addition, the total sample was also divided into three groups according to the age of the participants. The age ranges for the three groups were 20–35, 36–50, and 51–70. fNIR measures recorded from the optodes were grouped into four separate quadrants: lateral left (optodes 1, 2, 3, and 4), rostral left (optodes 5, 6, 7, and 8), rostral right (optodes 9, 10, 11, and 12), and lateral right (optodes 13, 14, 15, and 16). Each area was obtained from the mean of the four corresponding channels (Figure 1A). Pearson’s correlation analysis was performed to analyze the correlation between psychometric variables and prefrontal oxygenation. ANOVA and *t*-test analyses were performed to compare the levels of prefrontal oxygenation during the viewing of neutral and traffic accident-related pictures in the function of age, driving styles, or gender. Cohen’s d and eta square were calculated to assess effect size. According to the cut-off points indicated [45], a Cohen’s *d* value of 0.20 would be a small effect, 0.50 would be medium, and 0.80 would be large, whereas *η^2^* = 0.01 indicates a small effect, *η^2^* = 0.06 indicates a medium effect, and *η^2^* = 0.14 indicates a large effect.

## 3. Results

The analysis of the semantic differentials corresponding to the image blocks used revealed that the blocks containing images of traffic accidents had a valence rating of 28.56 points on a scale from 0 to 100 and an arousal rating of 68.73 points. On the other hand, the blocks with neutral images had a valence rating of 51.87 points and an arousal rating of 30.84 points.

Table 1 presents the means and standard deviations of age, MDSI scales, the factor obtained from the MDSI, and the oxygenation of the four quadrants of the prefrontal cortex for each gender. It also includes the observed differences between males and females, which were significant only for age (*t*-test = 3.3; *p* < 0.001; Cohen’s *d* = 0.46) and the Reckless scale (*t*-test = 2.28; *p* < 0.024; Cohen’s *d* = 0.32). Additionally, the table displays the values of Cronbach’s alpha for the MDSI scales. Women rated traffic accident images (*t*-test = 2.43; *p* < 0.016; Cohen’s *d* = 0.35) and neutral images (*t*-test = 2.19; *p* < 0.030; Cohen’s *d* = 0.30) with lower valence than men, while differences in arousal were only observed for traffic accident images (*t*-test = −3.06; *p* < 0.002; Cohen’s *d* = −0.44).

**Figure 2:**
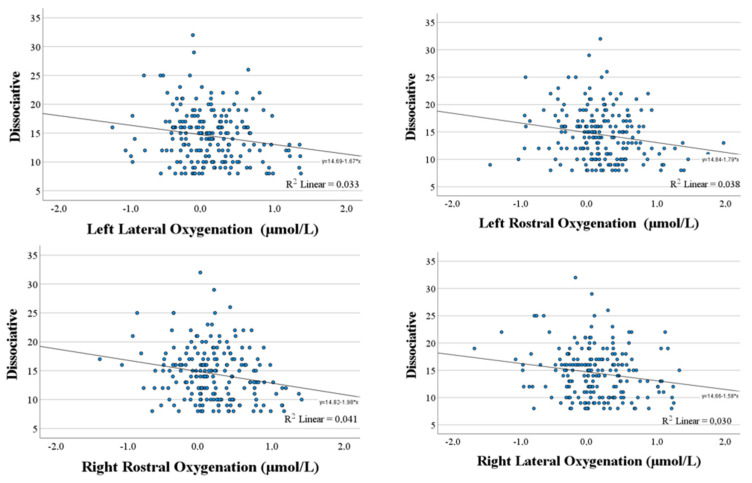
Relationships between Dissociative scale scores and oxygenation level changes (μmol/L) in each quadrant of prefrontal cortex.

Correlational analysis between psychometric variables and total oxygenation levels in each quadrant indicated an inverse relationship between Dissociative scale scores and oxygenation in all quadrants analyzed (all *p* ≤ 0.013; Figure 2). Moreover, a tendency for an inverse relationship was also observed between Anxious scale score and oxygenation in the right rostral area (*r* = −0.12; *p* < 0.09); although the other correlations were negative, they were not significant. No further significant correlations were found, although Careful scores were positively correlated with oxygenation levels, and Distress Reduction scores were also positively correlated, except for the right lateral area. In contrast, the Reckless scale showed negative correlations in all four quadrants.

**Figure 3:**
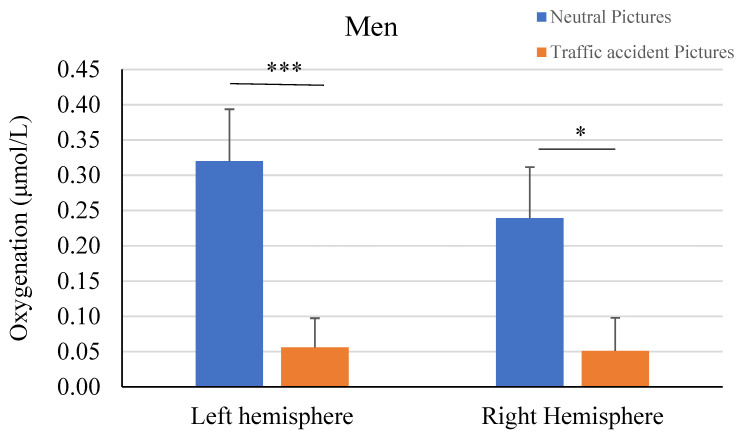

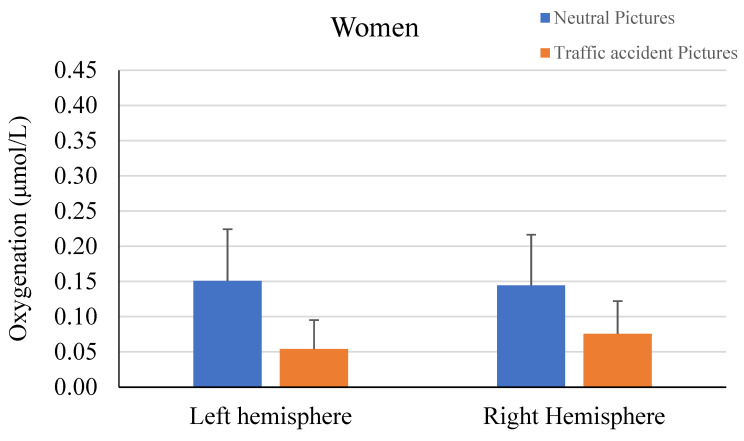
Differences in oxygenation of the prefrontal cortex viewing neutral and traffic accident pictures in men and women (* *p* < 0.05; *** *p* < 0.005).

When viewing neutral images, greater prefrontal oxygenation was observed compared to viewing images of traffic accidents. The left hemisphere (*t*-test = 3.23; *p* < 0.001; Cohen’s *d* = 0.23) showed greater differences than the right hemisphere (*t*-test = 2.46; *p* < 0.015; Cohen’s *d* = 0.17). Additionally, the left (*t*-test = 3.29; *p* < 0.001; Cohen’s *d* = 0.24) and right (*t*-test = 2.50; *p* < 0.013; Cohen’s *d* = 0.18) lateral areas exhibited greater differences than the left (*t*-test = 2.80; *p* < 0.006; Cohen’s *d* = 0.20) and right (*t*-test = 1.96; *p* < 0.052; Cohen’s *d* = 0.14) rostral areas. Separate analysis by gender indicated that these differences were attributable to the response of male participants (Figure 3). Once again, the differences were more significant in the lateral (*t*-test = 3.02; *p* < 0.003; Cohen’s *d* = 0.28) and rostral (*t*-test = 3.04; *p* < 0.003; Cohen’s *d* = 0.28) areas of the left hemisphere compared to the right rostral (*t*-test = 1.88; *p* < 0.063; Cohen’s *d* = 0.18) and lateral (*t*-test = 2.35; *p* < 0.021; Cohen’s *d* = 0.22) areas. In contrast, the analysis in the female sample showed no significant differences in either the left (*t*-test = 1.14; *p* < 0.26) or right (*t*-test = 0.95; *p* < 0.36) hemisphere.

**Figure 4:**
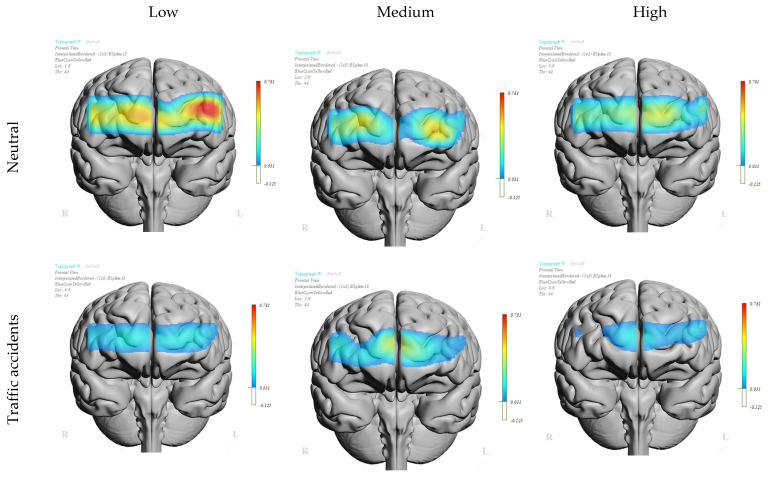
Differences in oxygenation of the prefrontal cortex viewing neutral and traffic accident pictures in low, medium, and high maladaptive style groups measured by the MDSI.

Figure 4 presents the topographic representation of the mean oxygenation levels for the sixteen channels of the neutral image blocks and the traffic accident image blocks for each of the three groups obtained from the single factor score of MDSI. A gradation in oxygenation levels can be observed. There is a noticeable variation in oxygenation levels among the groups. Individuals with a lower driving style factor score, indicating a more adaptive driving style, tended to exhibit higher oxygenation levels (0.17 μmol/L) during both the neutral and accident image blocks. Those with a medium driving style factor score showed a lower oxygenation level (0.14 μmol/L), and individuals with a higher driving style factor score, indicating a more maladaptive driving style, demonstrated the lowest oxygenation levels (0.11 μmol/L). Nevertheless, these differences between groups are not statistically significant. In contrast, when comparing oxygenation levels during the neutral and traffic accident imaging blocks within each group, significant differences are observed. Among drivers with more adaptive styles, the differences in oxygenation levels between neutral and accident blocks are evident in both the left (*t*-test = 2.72, *p* < 0.009; Cohen’s *d* = 0.34) and right hemispheres (*t*-test = 2.22 *p* < 0.030; Cohen’s *d* = 0.28). Similarly, drivers with a more maladaptive style display greater differences in oxygenation levels between the neutral and accident pictures in the rostral quadrants, reaching significance only in the left rostral area (*t*-test = 2.25, *p* < 0.028; Cohen’s *d* = 0.28). No significant differences were found regarding the assessment of valence and arousal between any of the three driving style subgroups (all *p* > 0.50). On the other hand, the comparison of means according to the scores on the Dissociative scale (low ≤ 12; medium 13–16; high ≥ 17) shows the existence of significant differences in men in the right rostral area *(F* = 3.28; *p* < 0.041; *η^2^* = 0.054). A tendency towards significance appeared in the right lateral area *(F* = 2. 51; *p* < 0.086; *η^2^* = 0.042). The highest levels of oxygenation were found in all quadrants in participants with lower scores on the Dissociative scale, while the lowest levels of oxygenation were observed in participants with higher scores on the Dissociative scale. No significant differences were found in females. A separate analysis of neutral imagery in men shows significant differences for neutral imagery in the right rostral area *(F* = 4.19; *p* < 0.018; *η^2^* = 0.07). In contrast, the analysis of the prefrontal response to accident images shows the existence of significant effects in the left *(F* = 4.23; *p* < 0.017; *η^2^* = 0.069) and right *(F* = 4.57; *p* < 0.012; *η^2^* = 0.074) ventral areas, but on this occasion, the highest levels of oxygenation are observed in those participants with average scores on the Dissociative scale.

Furthermore, we found an inverse relationship between age and scores obtained on the Anxious scale (*r* = −0.15; *p* < 0.035), Angry scale (*r* = −0.20; *p* < 0.005), and the factor derived from the MDSI scales (*r* = −0.15; *p* < 0.031). Nevertheless, we did not find any significant association between age and prefrontal oxygenation levels (all *p* > 0.42) or image assessment (all *p* > 0.49).

## 4. Discussion

This study investigated changes in prefrontal oxygenation during the viewing of neutral and traffic accident-related images among a heterogeneous cohort of drivers in an examination of their driving styles. Findings revealed that male drivers exhibiting a dissociative driving pattern displayed diminished prefrontal oxygenation levels during both neutral and accident-related imagery. Furthermore, prefrontal oxygenation was higher during neutral image viewing compared to accident images.

A notable observation in our study is the presence of gender disparities in prefrontal activation. Extensive research has delineated differences between males and females in prefrontal cortex development and function [46,47]. In addition, Hancock et al. [17] found gender differences in emotional regulation in response to task- or emotion-focused coping strategies. Our study revealed no significant effects in the analysis of female participants. Conversely, analysis of male participants revealed significant effects, albeit of modest to moderate magnitude. No gender discrepancies were observed in either driving style, except for the Reckless scale, or oxygenation levels. Several factors may account for the absence of significant differences in women, among which the effect of hormonal fluctuations characteristic of the menstrual cycle [48,49] and variances in the degree of cerebral lateralization [46,50] are noteworthy. On the basis of the results obtained, it was conjectured that drivers with maladaptive driving styles or elevated scores on related scales would display diminished prefrontal oxygenation. While our results supported this hypothesis, it was primarily associated with scores on the Dissociative scale and manifested variances by gender.

During the development of the MDSI, the Dissociative scale accounted for a greater percentage of the variance (21%) and characterized drivers with high scores as having a tendency “to be easily distracted during driving, to commit driving errors due to this distraction, and to display cognitive gaps and dissociations during driving” [7]. This conduct might stem from an inability to regulate attention. Three networks implicated in the attentional system have been identified: alerting, orienting, and executive [51,52]. The executive attentional system entails prefrontal cortex activation through a cognitive network or a dual mechanism. In the former case, the dorsolateral prefrontal cortex would exert top-down inhibition on the basis of performance information provided by the medial areas, whereas in dual networks, these systems would operate independently [52]. Participants with higher Dissociative scores exhibited reduced activity in the right prefrontal cortex during neutral image viewing, potentially indicating poorer attentional control. Indeed, activation of the right prefrontal cortex is associated with vigilance and sustained attention [53], and its stimulation improves attentional control [54]. It has also been theorized that poor right prefrontal functioning could be responsible for the inability to discriminate between neutral and emotional images in patients with depression [55]. Conversely, greater right prefrontal activation in men suggested higher attentional capacity and emotional discrimination, contributing to a more adaptive driving style.

Regarding the response of ventral areas to accident images, an inverted U-shaped effect was observed, with average Dissociative scorers showing higher oxygenation levels. This scale would have been linked to anxious personality traits or neuroticism [56,57], which is associated with the response to emotional arousal [58], presenting an inverted U-shaped relationship with cognitive performance [59]. Functional connectivity studies highlighted negative correlations between neuroticism and both the amygdala and the left lateral orbitofrontal and ventrolateral PFC [60]. A greater sensitivity of the ventral prefrontal cortex to the intensity of the emotional stimulus than to its valence has been shown [61]. Additionally, lateral and medial prefrontal areas demonstrated sensitivity to external and internal emotional changes, respectively [62].

Another noteworthy aspect is the differences in prefrontal oxygenation levels as a function of image valence. The findings indicated higher oxygenation levels during neutral image viewing in all prefrontal areas. Different relationships between image valence and neural activity have been observed. In dorsal prefrontal areas, an inverted U-shaped relationship between image valence and neural activity has been described [63]. This implies that the highest levels of neuronal activation would occur with images with neutral valence, as we can observe in our study. Although significant differences were detected in all prefrontal areas analyzed, the most notable effects emerged in the left hemisphere. This may be attributed to hemispheric differences in emotional response [64]. Lastly, despite finding no differences based on age, an inverse relationship between age and maladaptive driving style was observed. This finding aligns with prior research [65].

Nonetheless, this study is not without limitations. Firstly, although the driving style factor correlated well with the number of reported complaints, additional measures, such as years of experience or annual kilometers driven, might have been useful. Moreover, the use of driving simulators could provide us with more direct information on the participant’s driving style. Also, bearing in mind the importance of personality characteristics in the emotional impact of the images, the availability of personality measures could have been helpful. Additionally, the unequal sample sizes of men and women may introduce bias. It is therefore imperative to delve deeper into the identified gender differences. Furthermore, while menstrual cycle information was gathered from female participants, further control of the menstrual cycle phase, including hormonal assessments, could enhance the study’s findings.

Notwithstanding its limitations, this study boasts several strengths. It was conducted with a sizable sample size, encompassing both genders and a wide age range. Additionally, the utilization of traffic accident-related images allowed for the assessment of participants’ emotional responses directly related to the characteristic being studied. Lastly, the use of spectroscopy facilitated the evaluation of prefrontal cortex response in a comfortable setting for participants, circumventing interference from discomfort prevalent in other neuroimaging techniques.

## 5. Conclusions

In conclusion, our study suggests lower prefrontal oxygenation levels in male drivers with maladaptive driving styles, particularly dissociative ones, in response to neutral and traffic accident images, whereas this response was not evident in female drivers, nor did significant differences emerge based on age.

## Figures and Tables

**Table 1:** Means and standard deviations for each gender of age, MDSI scales, the factor obtained from the MDSI, and the oxygenation of the four quadrants of the prefrontal cortex and gender differences.

		Men		Women				
		Mean	SD	Mean	SD	*t*	*p*<	α
	Age	44.38	12.98	38.89	10.60	3.30	0.001	
MDSI	Reckless	14.47	6.81	12.48	5.56	2.28	0.024	0.88
	Anxious	9.08	4.19	8.98	4.14	0.17	0.867	0.80
	Careful	32.81	4.63	32.79	6.48	0.04	0.972	0.80
	Angry	8.57	3.47	8.73	3.47	−0.32	0.749	0.77
	Dissociative	14.47	4.46	14.55	4.63	−0.11	0.910	0.71
	Distress Reduction	17.05	3.15	17.04	3.84	0.03	0.976	0.67
	MDSI Factor	0.05	0.99	−0.07	1.01	0.87	0.384	
Oxygenation Change	Lateral Left	0.15	0.47	0.06	0.53	1.23	0.221	--
	Rostral Left	0.21	0.52	0.14	0.46	0.92	0.359	--
	Rostral Right	0.18	0.48	0.16	0.44	0.30	0.764	--
	Lateral Right	0.12	0.52	0.06	0.48	0.78	0.438	--

## Data Availability

The authors declare that the use of these data in a public repository is prohibited until the project is completed in Spain. However, some minor data about this manuscript may be requested from the first author if there is a non-dissemination commitment.
